# Endometrial thickness is not a risk factor for hypertensive disorders of pregnancy after IVF treatment in women with male-factor infertility

**DOI:** 10.3389/fendo.2026.1896764

**Published:** 2026-07-17

**Authors:** Ningning Chen, Xiaopei Guo, Junjie Zhao, Wei Shi, Xiaohua Luo

**Affiliations:** Department of Obstetrics and Gynecology, The Third Affiliated Hospital of Zhengzhou University, Zhengzhou, China

**Keywords:** endometrial thickness, frozen-thawed embryo transfer, hypertensive disorders of pregnancy, *in vitro* fertilization, male factor, retrospective cohort study

## Abstract

**Background:**

The relationship between endometrial thickness and adverse pregnancy outcomes has garnered significant attention from researchers. The aim of this study was to investigates whether endometrial thickness (EMT) is a risk factor for hypertensive disorders of pregnancy (HDP) in women undergoing *in vitro* fertilization (IVF) due to male-factor infertility.

**Methods:**

This single center retrospective cohort study included 2,536 women aged 20–40 years with male-factor infertility who underwent their first frozen-thawed embryo transfer (FET) and had a singleton live birth. Endometrial thickness was measured via transvaginal ultrasound on the day of embryo transfer, and patients were divided into three groups: ≤8 mm, 8–14 mm, and ≥14 mm. The primary outcome was the incidence of HDP.

**Results:**

The overall HDP rate was 3.67%, with no significant difference across EMT groups: 3.97% in the ≤ 8 mm group, 3.74% in the 8–14 mm group, and 3.03% in the ≥ 14 mm group (p=0.760). Older maternal age and higher BMI were significant risk factors for HDP [maternal age (OR 1.10, 95% CI 1.05-1.16, p=0.000) and BMI ≥ 24.0 Kg/m² (OR 2.40, 95% CI 1.10-5.25, p=0.028)], while EMT was not associated with HDP risk.

**Conclusion:**

Endometrial thickness was not a significant risk factor for HDP in women with male-factor infertility undergoing their first FET.

## Introduction

Since the success of the world’s first *in vitro* fertilization and embryo transfer (IVF-ET) treatment, an increasing number of infertile women have achieved pregnancy with this technology. Endometrial receptivity and embryonic factors are the foundation IVF success. With the continuous advancement of science and technology, methods such as Doppler ultrasound and endometrial biopsy have made it more convenient and effective to assess endometrial receptivity (1, 2). However, due to its non-invasive, intuitive, convenient, and cost- effective nature, Doppler ultrasound has been widely applied in clinical practice in reproductive medicine. Currently, numerous scholars have utilized Doppler ultrasound to measure the correlation between endometrial thickness and pregnancy outcomes (3–5).

The relationship between endometrial thickness and adverse pregnancy outcomes has garnered significant attention from researchers. Previous studies have shown that in frozen-thawed embryo transfer cycles, thin endometrium is significantly negatively correlated with clinical pregnancy rates and increases the risk of miscarriage and ectopic pregnancy (6, 7). A recent meta-analysis including 22 studies indicated that in fresh transfer cycles, endometrial thickness on the day of hCG administration is related to clinical pregnancy rates and live birth rates, with the thin endometrium group also showing a significantly increased risk of low birth weight infants (8). In 2014, a study involving 4,537 singleton newborns found that following IVF, the risk of placenta previa is increased 4-fold in women with an endometrial thickness of >12 mm compared with women with an endometrial thickness of <9 mm (9).

Hypertensive disorders of pregnancy (HDP) are conditions where a pregnant woman develops high blood pressure, posing risks to both mother and baby (10). These include gestational hypertension, preeclampsia, eclampsia, and chronic hypertension. HDP can lead to serious complications like preterm birth, low birth weight, or placental abruption. Recently, a retrospective study involving 2,275 singleton live births demonstrated a noteworthy association between endometrial thickness (EMT) and adverse perinatal outcomes during programmed frozen-thawed embryo transfer (FET) cycles (11). Specifically, a thick endometrium (EMT >12 mm) was independently associated with an increased risk of developing HDP, whereas the optimal EMT for reducing the risk of HDP was around 9–10 mm. However, other studies have shown that thin endometrium is an independent risk factor for HDP (12).

Since the occurrence of HDP is influenced by various factors, including maternal age, number of pregnancies, obesity, etc., these factors may act individually or collectively to increase the risk of HDP. Therefore, to clarify the relationship between endometrial thickness on the day of transfer in frozen-thawed cycles and HDP, this study only included first frozen-thawed cycles of IVF due to male factors, aiming to explore the impact of endometrial thickness on HDP and provide guidance for clinical practice.

## Materials and methods

This retrospective cohort study included patients who achieved pregnancy via FET and subsequently delivered a singleton liveborn infant at the Department of Obstetrics, Third Hospital of Zhengzhou University, between January 1, 2016, and December 31, 2022. The endometrial thickness of the patients on the day of embryo transfer was measured and recorded using transvaginal ultrasound. Meanwhile, general clinical data, laboratory data, clinical pregnancy outcomes, pregnancy complications, and live birth outcomes were collected from the electronic medical record system in our hospital. All patients were informed and signed informed consent forms, and this study was approved by the Ethics Committee of the Third Affiliated Hospital of Zhengzhou University.

Inclusion Criteria were as follows: female age between 20 and 40 years old; IVF/ICSI cycles due to azoospermia/oligospermia; first frozen-thawed embryo transfer; singleton live birth (including spontaneous reduction of twins). Exclusion Criteria were: preimplantation genetic testing cycles; uterine malformations, submucosal fibroids, or endometrial polyps; pre-existing chronic hypertension, diabetes, thyroid disorders, or hyperprolactinemia; oocyte donation cycles; polycystic ovary syndrome and other additional female factors were excluded from the study. In addition, in order to exclude surgery history of uterine, patients with miscarriage or cesarean section were also not included.

According to the 2022 ASRM Clinical Practice Guidelines and the 2023 ESHRE ART Consensus Recommendations, patients were divided into three groups based on endometrial thickness on the day of transplantation: Group A (≤8 mm), Group B (>8–14 mm), and Group C (≥14 mm).

### Endometrial preparation protocols

Natural Cycle: For patients with regular menstrual cycles, follicular development and endometrial status were monitored via ultrasound starting from days 9–10 of the menstrual cycle. After confirming ovulation, progesterone injections (60 mg/day, Zhejiang Xianju Pharmaceutical) were administered intramuscularly, and embryo transfer was performed 4–5 days later.

Artificial Cycle: For patients with irregular menstrual cycles, estradiol valerate tablets (Progynova, 1 mg/tablet, Bayer) were administered starting from day 3 of the menstrual cycle after ultrasound confirmed no significant abnormalities in the uterus or adnexa. The dosage was adjusted based on endometrial changes. When a clear trilaminar endometrial pattern was observed on ultrasound on days 14–16, progesterone injections and dydrogesterone tablets (Duphaston, 10 mg/tablet, Abbott) were administered, and embryo transfer was scheduled 5–6 days later.

### Embryo transfer and luteal support

Embryos were frozen and thawed using the rapid vitrification method. Embryo survival was defined as more than 50% of cells surviving after thawing. One to two high-quality embryos were routinely transferred per cycle. Starting the day after embryo transfer, patients received intravaginal progesterone gel (Crinone, 90 mg/tube, Merck) and oral dydrogesterone for luteal support. Blood hCG levels were measured on days 14 and 18 post-transfer. If hCG was positive on day 18, an ultrasound was performed on day 35 to confirm intrauterine pregnancy.

The primary outcome was HDP, including gestational hypertension, pre-eclampsia, eclampsia, chronic hypertension complicating pre-eclampsia, as well as chronic hypertension complicating pregnancy. Only the first three categories were included in our study. According to the 2020 Chinese Guidelines for Hypertensive Disorders of Pregnancy, the diagnostic criteria for gestational hypertension are defined as a blood pressure reading of ≥ 140/90 mmHg, measured at least twice with an interval of more than 4 hours, in pregnant women who previously had normal blood pressure. Preeclampsia is characterized by the new onset of systemic complications on the basis of gestational hypertension, including proteinuria (24-hour urinary protein ≥ 300 mg or a urine protein/creatinine ratio ≥0.30), blurred vision, and abnormal liver or kidney function. Additionally, eclampsia refers to the occurrence of seizures in a pregnant woman with preeclampsia without any other precipitating factors, and it is also one of the severe manifestations of HDP.

### Statistical analysis

All data in this study were statistically analyzed using IBM SPSS Statistics 23.0. The Shapiro- Wilk test was used to assess whether numerical variables conformed to a normal distribution. Numerical variables that followed a normal distribution were expressed as mean ± standard deviation (X ± SD). Categorical variables were expressed as percentages (%). Comparisons of continuous variables among the three groups were performed using one-way ANOVA. Chi- square analysis or Fisher’s exact probability test was used to examine differences in proportions of categorical variables between two or more groups. Logistic regression was employed to analyze risk factors for HDP, with a P < 0.05 considered statistically significant.

## Results

This study included 2,536 singleton births following the first frozen-thawed embryo transfer in women with male-factor infertility. As shown in **Table 1**, the mean age of the women was 28.99 ± 3.93 years, with an average infertility duration of 4.02 ± 3.20 years. The mean endometrial thickness on the day of embryo transfer was 10.55 ± 1.93 mm, with 11.90% of women having an endometrial thickness ≤ 8 mm, 73.80% between 8–14 mm, and 14.30% ≥ 14 mm. The overall rate of HDP was 3.67% (93/2,536).

**Table 1:** Basic characteristics of the 2,536 singleton births after first frozen-thawed treatment in women with male-factor infertility.

Parameters	*N* = 2,536
Female age (years)	28.99 ± 3.93
Infertility duration (years)	4.02 ± 3.20
Gravidity	0.25 ± 0.43
No. of miscarriage	/
Basic FSH (mIU/mL)	6.56 ± 2.64
Basic AMH (ng/mL)	5.01 ± 3.54
BMI (Kg/m^2^)	22.48 ± 3.19
≤ 19.5 Kg/m^2^	17.00% (431/2,536)
19.6-23.9 Kg/m^2^	55.10% (1,397/2,536)
≥ 24.0 Kg/m^2^	27.90% (708/2,536)
Endometrial thickness on day of embryo transfer	10.55 ± 1.93
≤ 8 mm	11.90% (302/2,536)
8–14 mm	73.80% (1,871/2,536)
≥ 14 mm	14.30% (363/2,536)
Endometrial preparation protocol	
Estrogen-progesterone	60.20% (1,527/2,536)
Natural cycle	39.80% (1,009/2,536)
Type of embryos transferred	
Cleavage stage	51.80% (1,314/2,536)
Blastocyst	48.20% (1,222/2,536)
No. of embryos transferred	1.60 ± 0.49
HDP rate (%)	3.67% (93/2,536)

FSH, Follicle-Stimulating Hormone; AMH, Anti-Müllerian Hormone; BMI, Body Mass Index; HDP, Hypertensive Disorders of Pregnancy. Data were Mean ± SD unless otherwise noted.

Patients were stratified into three groups based on endometrial thickness (EMT): ≤ 8 mm, 8–14 mm, and ≥ 14 mm, as shown in **Table 2**. Women with EMT ≥ 14 mm were slightly older (29.70 ± 4.11 years) compared to those with EMT ≤ 8 mm (28.83 ± 3.98 years) and 8–14 mm (28.88 ± 3.88 years). However, the HDP rate was similar across all EMT groups: 3.97% in the ≤ 8 mm group, 3.74% in the 8–14 mm group, and 3.03% in the ≥ 14 mm group (p=0.760).

**Table 2:** Patient characteristics and HDP in three groups according to endometrial thickness.

Parameters	EMT ≤ 8mm	8 < EMT <14 mm	EMT ≥ 14 mm	F/χ^2^	P
N	302	1,871	363		
Female age (years)	28.83 ± 3.98	28.88 ± 3.88	29.70 ± 4.11	6.86	0.001
Infertility duration (years)	3.87 ± 3.03	4.03 ± 2.85	4.08 ± 4.72	0.37	0.693
Gravidity	0.26 ± 0.44	0.23 ± 0.42	0.31 ± 0.46	4.32	0.013
No. of miscarriage	/	/	/		
Basic FSH (mIU/mL)	6.55 ± 3.02	6.51 ± 2.60	6.81 ± 2.64	2.43	0.088
Basic AMH (ng/mL)	5.81 ± 4.37	5.02 ± 3.44	4.31 ± 3.14	8.45	0.000
BMI (Kg/m^2^)	22.75 ± 3.04	22.41 ± 3.22	22.62 ± 3.21	1.84	0.159
Endometrial preparation protocol	N =302	N =1,871	N =363	100.45	0.001
Estrogen-progesterone	72.52% (219/302)	61.36% (1,148/1,871)	44.08% (160/363)		
Natural cycle	27.48% (83/302)	38.64% (723/1,871)	55.92% (203/363)		
Type of embryos transferred				6.78	0.034
Cleavage stage	47.02% (142/302)	52.43% (981/1,871)	52.62% (191/363)		
Blastocyst	52.98% (160/302)	47.57% (890/1,871)	47.38% (172/363)		
No. of embryos transferred	1.60 ± 0.49	1.60 ± 0.49	1.60 ± 0.49		
HDP rate (%)	3.97% (12/302)	3.74% (70/1,871)	3.03% (11/363)	0.55	0.760

EMT, endometrial thickness; FSH, Follicle-Stimulating Hormone; AMH, Anti-Müllerian Hormone; BMI, Body Mass Index; HDP, Hypertensive Disorders of Pregnancy. Data were Mean ± SD unless otherwise noted.

In univariate logistic regression analysis, older maternal age (OR 1.10, 95% CI 1.05-1.16, p< 0.001) and higher BMI (≥ 24.0 Kg/m², OR 2.92, 95% CI 1.35-6.32, p=0.007) were significant risk factors for HDP. The use of a natural cycle for endometrial preparation was associated with a lower risk of HDP compared to estrogen-progesterone (OR 0.61, 95% CI 0.39-0.96, p=0.032). In multivariate analysis, maternal age (OR 1.10, 95% CI 1.05-1.16, p< 0.001) and BMI ≥ 24.0 Kg/m² (OR 2.40, 95% CI 1.10-5.25, p=0.028) remained significant risk factors for HDP. However, as shown in **Table 3**, endometrial thickness was not significantly associated with HDP risk in either univariate or multivariate analyses.

**Table 3:** Risk factor for HDP in univariate and multivariate logistic regression analysis.

Parameters	Univariate			Multivariate		
	OR	95% CI	*P*	OR	95% CI	*P*
Age	1.10	1.05-1.16	0.000	1.10	1.05-1.16	0.000
Infertility type						
Primary	1.00					
Secondary	1.31	0.84-2.05	0.242			
BMI (Kg/m^2^)						
≤ 19.5 Kg/m^2^	1.00			1.00		
19.6-23.9 Kg/m^2^	1.88	0.88-4.01	0.101	1.68	0.78-3.59	0.182
≥ 24.0 Kg/m^2^	2.92	1.35-6.32	0.007	2.40	1.10-5.25	0.028
Endometrial preparation protocol						
Estrogen-progesterone	1.00			1.00		
Natural cycle	0.61	0.39-0.96	0.032	0.70	0.45-1.05	0.057
Type of embryos transferred						
Cleavage stage	1.00					
Blastocyst	1.55	0.99-2.40	0.147			
No. of embryos transferred	1.17	0.77-1.78	0.453			
Endometrial thickness						
≤ 8 mm	1.32	0.58-3.05	0.509	1.28	0.55-2.98	0.565
8–14 mm	1.24	0.65-2.37	0.508	1.29	0.67-2.49	0.441
≥ 14 mm	1.00			1.00		

BMI, Body Mass Index; HDP, Hypertensive Disorders of Pregnancy; OR, odds ratio; CI, confidence interval.

## Discussion

HDP is a group of conditions characterized by the coexistence of hypertension and pregnancy. Globally, the incidence of HDP ranges from 2% to 8%, making it a significant threat to maternal and fetal safety during the perinatal period (13, 14). Research into the treatment of HDP has never ceased, yet, apart from delivery, no effective method to halt the progression of the disease has been identified in clinical practice. To enhance the management of high-risk pregnant women and improve the clinical outcomes of patients with HDP, exploring the risk factors for HDP is of paramount importance.

In 2019, He et al. conducted a study involving 1,139 patients who underwent *in vitro* fertilization and embryo transfer (IVF-ET). They found that in the thin endometrium group, the risks of premature rupture of membranes and postpartum hemorrhage were significantly higher compared to other endometrial thickness groups, but the incidence of HDP did not differ significantly across endometrial thickness groups (15). However, a subsequent larger retrospective study in 2021 found that, after controlling for other confounding factors, thin endometrium remained an independent risk factor for HDP (12). The exact mechanisms by which thin endometrium influences HDP remain unclear, but here are several potential mechanisms: Thin endometrium is often associated with embryo implantation and endometrial decidualization. Studies suggest that insufficient endometrial thickness may lead to reduced endometrial receptivity, resulting in failed embryo implantation or poor embryonic development. Endometrial thickness is not only a physical barrier but also reflects endometrial blood flow, stromal thickness, and glandular secretory function (16). These factors may collectively affect placental formation and development, and poor placental development is one of the key pathophysiological bases of HDP (17). Meanwhile, some studies indicate that thin endometrium may be associated with insufficient endometrial blood flow. Abnormal endometrial blood flow can affect embryonic nutrient supply, thereby impacting placental development and leading to placental hypoperfusion. Placental hypoperfusion can activate the maternal renin-angiotensin system and sympathetic nervous system, resulting in elevated blood pressure and the onset of HDP (18). Additionally, insufficient endometrial blood flow may be related to high resistance in the uterine arteries, which is another important pathological feature of HDP (19).

After embryo implantation, the endometrium in contact with the embryo forms the decidua basalis, which constitutes the maternal portion of the placenta (20). Therefore, the thickness and morphology of the endometrium at the time of embryo implantation determine placental function and influence placental-related maternal and fetal complications, including placenta previa, placental abruption, and HDP caused by placental vascular pathology (21). However, existing literature argues that most patients with thin endometrium have a history of intrauterine procedures and often suffer from conditions such as intrauterine adhesions and adenomyosis (22). These conditions can affect endometrial thickness and morphology, thereby impacting embryo implantation, development, and subsequent outcomes. The question remains: is thin endometrium itself closely related to HDP, or do the underlying conditions of patients with thin endometrium lead to an increased incidence of HDP? To address this, our study focused exclusively on patients undergoing IVF/ICSI due to male-factor infertility and excluded those with a history of abortion or cesarean section. The results showed no significant relationship between endometrial thickness and the occurrence of HDP during pregnancy. The findings from this study provide important insights for patients undergoing FET with male- factor infertility. As expected, patients who are older or have a higher BMI should be particularly vigilant about monitoring their blood pressure and overall health during pregnancy. However, patients need not be overly concerned about EMT in relation to HDP risk, as it does not appear to be a major contributing factor.

Although our findings are inconsistent with most previous studies, our study used a model of patients with male-factor infertility, often referred to as “standard patients,” which reduces bias and provides more objective and reliable results. However, several limitations exist. as this is a single-center retrospective analysis, some unavoidable biases exist, and caution is needed when interpreting the results. Secondary outcomes such as severe preeclampsia and preterm birth were not followed up. Additionally, the inclusion of cycles involving testicular/epididymal sperm extraction may have an unclear impact on HDP.

In conclusion, these findings highlight the importance of individualized care, particularly for patients with advanced maternal age or elevated BMI. Importantly, patients can be reassured that endometrial thickness is not a significant factor in HDP risk, allowing them to focus on other aspects of their health and pregnancy care. By understanding these risk factors and maintaining open communication with healthcare providers, patients can take proactive steps to optimize their pregnancy outcomes.

## Data Availability

The original contributions presented in the study are included in the article/supplementary material. Further inquiries can be directed to the corresponding authors.
